# Exploitation may influence the climate resilience of fish populations through removing high performance metabolic phenotypes

**DOI:** 10.1038/s41598-019-47395-y

**Published:** 2019-08-07

**Authors:** Murray I. Duncan, Amanda E. Bates, Nicola C. James, Warren M. Potts

**Affiliations:** 1 Department of Ichthyology and Fisheries Science, Rhodes University, Grahamstown, 6140 South Africa; 2 South African Institute for Aquatic Biodiversity, Grahamstown, 6139 South Africa; 3 Department of Ocean Sciences, Memorial University of Newfoundland, St. John’s, A1C 5S7 Canada

**Keywords:** Ecophysiology, Metabolism

## Abstract

Physiological rates and processes underpin the relationships between ectothermic organisms, such as fish, and their environment. The response and persistence of fish populations in an increasingly variable ocean is dependent on the distribution and diversity of physiological phenotypes. Growing evidence suggests that fisheries exploitation can selectively target certain physiological and behavioural phenotypes, which may shift exploited populations to altered physiological states. Here we test if commercial fisheries have the potential to do this in a “natural laboratory” along the South African coast. We compare metabolic traits of exploited and protected populations of the fish species, *Chrysoblephus laticeps*, which is a major component of the South African hook and line fishery. We find that high-performance aerobic scope phenotypes are reduced in the fished population. The most likely mechanism for this finding is a positive relationship between aerobic scope and capture vulnerability in passive-gear fisheries. Our results further highlight the selective nature of capture-fisheries and suggest that exploitation has the capacity to alter climate responses of fish populations on a physiological level. Our finding also implicates how Marine Protected Areas, through harbouring individuals with a greater diversity of physiological traits, may provide greater fish response diversity to environmental variability.

## Introduction

Wild fish populations are increasingly challenged to persist in an ocean that is being reshaped by rapidly increasing temperatures, acidification, expansion of oxygen dead zones, and higher frequencies and magnitudes of extreme weather events^1–5^. At the same time, selective fisheries exploitation may be compromising the capacity of fish populations to resist or recover from climate disturbances, thus reducing their overall resilience^6,7^. Fishing often targets individuals with certain traits including large body sizes, faster growth rates and bold/active behaviour types, among many others^8–10^. Trait selection can shift demographic, life history and trait diversities of exploited fish populations reducing their buffering capacity to climate disturbances^11,12^, such as the removal of large body sizes and their disproportionately higher fecundity^13^. Despite the role physiological traits play in modulating organism responses to climate^14,15^ there has been limited research into whether fisheries exploitation can alter the physiological trait distributions of wild populations^16^.

Fish are ectotherms, as such their physiological rates are governed by external temperatures^17^ and these physiological rates underpin many behaviour, phenology, demographic or distributional responses^18,19^. It is therefore alterations to physiological trait distributions that may have the greatest potential to move populations towards states that are less resilient to climate change^20,21^. Given the projected increases in the frequency and magnitude of temperature-variability as global climate change continues^2,22–24^, the capacity for fish populations to survive extreme temperature events may relate to their ability to maintain physiological processes, e.g.^25^. At the same time evidence is accumulating that a fish’s physiological state or phenotype may underpin vulnerability to capture, particularly in passive-gear fisheries^26–28^. Thus understanding how fisheries exploitation can alter the baseline physiology of fish populations is a pre-requisite to assess climate change resilience and formulate informed management strategies^29^.

Here we focus on fundamental physiological metrics of performance – metabolic rates. Metabolic rates describe the total amount of internal chemical reactions that creates, maintains or manipulates energy^30^ and is quantified as the rate of oxygen consumption^31^. Standard metabolic rate (SMR) represents the energetic requirements for maintenance^32^, while maximum metabolic rate (MMR) is the highest rate of aerobic energy conversions^33^. Absolute aerobic scope (MMR – SMR) therefore describes the capacity to fuel aerobic energetic process above that required to meet basal maintenance demands^34,35^ and can vary up to three-fold among conspecifics (metabolic phenotypes). The ability of an organism to raise metabolism above SMR levels may result in a performance advantage^35,36^, such that aerobic scope phenotypes can be classified from low to high performance phenotypes^37^, although the benefit of a high performance aerobic scope may be context dependant^38^ and see^39^ for evidence against this universal application.

**Figure 1:**
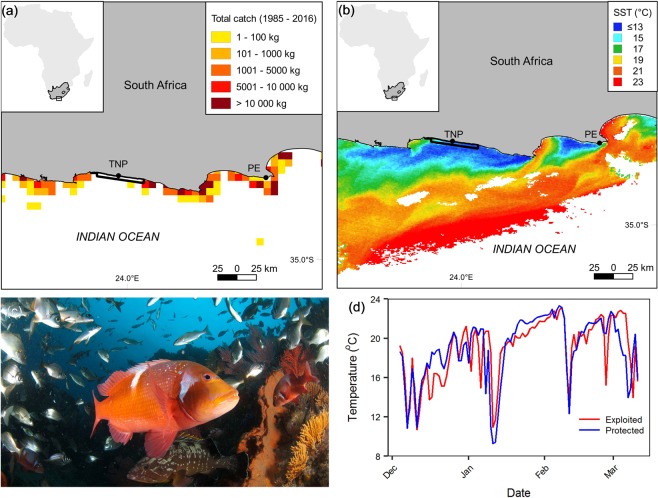
Study site contextualisation. (**a**) Location of exploited (west of Port Elizabeth - PE) and protected (within the Tsitsikamma National Park - TNP) sampling areas in relation to total commercial catch of *Chrysoblephus laticeps* between 1985 and 2016. (**b**) Upwelling events depicted by MODIS Terra satellite sea surface temperature data taken on 04-03-2010. (**c**) Photograph of Chrysophlephus laticeps on a typical reef in South Africa (Image credit: Steven Benjamin). (**d**) Synchrony of temperature-variability regimes between sampling areas generated from daily underwater temperature recorder (TNP = 10 m, PE = 5 m) sea temperature data from December 2002 to March 2003. The boundary of TNP is outlined in black.

**Figure 2:**
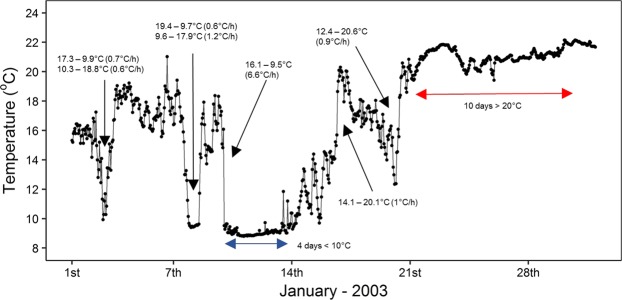
High rate of temperature change corresponds with experimental protocols. Hourly sea temperature data (19 m depth) (black points) from within the TNP during January 2003 with major upwelling/downwelling events indicated with black arrows highlighting range and rate of temperature change and anomalous cold (blue arrow) and hot (red arrow) spells.

Our overarching objective was to test whether selective fisheries exploitation can alter the distribution of metabolic phenotypic traits. To do so we compare metabolic traits of heavily exploited populations of the reef fish, *Chrysoblephus laticeps*, with populations that have been protected from fishing for over five decades within the ≈ 360 km^2^; Tsitsikamma National Park (TNP) Marine Protected Area (MPA) (Fig. 1). Our sites were carefully selected on the basis of similar thermal regimes, whereby both areas experience acute thermal variability with temperature fluctuations of 10 °C within a few hours in either direction a common occurrence (e.g. Fig. 2). Furthermore, *C. laticeps is* an exemplary focal species because it displays high site fidelity^40^, and has been protected within the TNP MPA for many decades, offering a baseline against which the effects of exploitation can be compared. We quantify metabolic phenotypic traits of individuals from protected and exploited populations based on standard metabolic rate, maximum metabolic rate and absolute aerobic scope across the full range of temperatures where this species is found and at rates of change that mimic observed and predicted acute temperature-variability at the locations in which fish were sampled.

## Results

### Metabolic rates and temperature relationships

**Figure 3:**
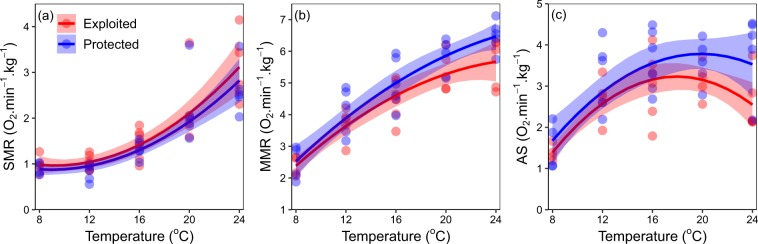
Physiological rates for exploited and protected populations. (**a**) Standard metabolic rate (SMR) (n = 50). (**b**) Maximum metabolic rate (MMR) (n = 49). (**c**) Absolute aerobic scope (AS) (n = 49) measured across test temperatures per exploited (red) and protected (blue) sampling areas. Individual data are indicated by points and the modelled second order polynomial relationship with temperature is the solid line with shaded 95% confidence intervals.

Standard metabolic rates increase logarithmically with temperature and are similar in both exploited and protected populations (Generalised Least Squares, GLS: *T* = −1.266, *p* > 0.05, Fig. 3a, Supplementary Material Table SM1a). While both maximum metabolic rate and absolute aerobic scope also scale positively with temperature, there is a divergence between the exploited and protected populations at warmer temperatures (Fig. 3b,c) for maximum metabolic rates (GLS: *T* = 2.53, *p* < 0.05, Supplementary Material Table SM1b) and aerobic scope (GLS: *T* = 2.85, *p* < 0.05, Supplementary Material Table SM1c). After a minimum of 42 days acclimation in similar laboratory conditions, individuals from exploited populations had significantly lower absolute aerobic scopes than reference protected populations across all temperatures tested (Fig. 3c).

**Figure 4:**
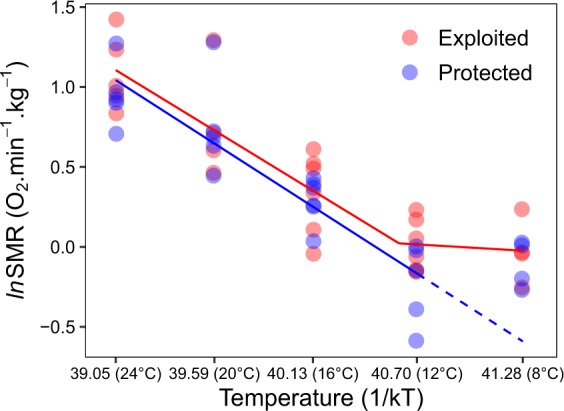
Standard metabolic rate and temperature relationship. Natural logarithm of standard metabolic rate (*ln*SMR) and the inverse product of temperature (Temperature is in Kelvin degrees) and the Boltzmann constant (k) for exploited (red) and protected (blue) populations. Corresponding test temperatures in degrees Celsius are indicated in brackets. The piecewise breakpoint relationship for the exploited population (breakpoint = 40.6 or 12.7 °C) (solid red line), linear relationship for the protected population (solid blue line) and predicted range of breakpoint (dashed blue line) are shown.

We further find that standard metabolic rate stopped scaling predictably with temperature at 12.7 °C for exploited populations (Fig. 4), indicated by piecewise linear breakpoint analysis. By contrast, the breakpoint could not be identified for individuals from the protected population at any of the test temperatures, suggesting protected individuals are able to maintain basal metabolic rates across a wider thermal gradient. Furthermore, metabolic phenotypic diversity and aerobic scope magnitude was reduced at the coldest temperature (8 °C) in individuals from both the exploited and protected populations (Fig. 3a–c).

**Figure 5:**
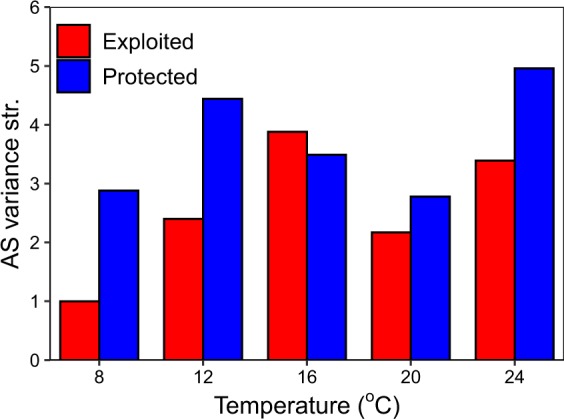
Aerobic scope phenotypic diversity. Variance structure of the modelled relationship between aerobic scope (AS variance str.) and temperature for exploited (red) and protected (blue) populations. There was significantly more variability in the protected population than the exploited population (paired one-way t-test; *p* < 0.05, *T* = −2.5, df = 4).

There is also less phenotypic variability in aerobic scope among individuals from exploited versus protected populations (paired one-way t-test; p-value = 0.033, t-stat = −2.5, df = 4) (Fig. 5). This higher phenotypic variability among protected populations is more pronounced at the warmest and coolest test temperatures, with little difference observed at 16 °C when the temperature matched the holding temperature (Fig. 5).

### Thermal and test specimen characteristics between study areas

Year-to-year differences in mean annual SST were similar in direction and magnitude (Supplementary Material Fig. SM1a) and the difference between the area-specific trend splines of the GAMM model (Supplementary Material Fig. SM1b, dashed black line with 95% CI shaded grey) did not differ from zero (*p*-value > 0.05, Supplementary Material Table SM2), indicating similar long-term sea temperature trends between both areas. The random component of the UTR time series data was strongly synchronous between areas (mean Pearson’s correlation: 0.572, *p*-value < 0.001), indicting a similar direction and magnitude of abrupt departures from mean sea temperatures at sampling locations (*p*-value < 0.05, Fig. 1d, Supplementary Material Fig. SM2a–c). We found no difference in mass or condition between individuals collected from each of the two areas (*p-value* > 0.05, Supplementary Material Table SM3, Fig. SM3a) or across temperature treatments between sampling areas (Supplementary Material Fig. SM3b).

## Discussion

### Exploitation, metabolic traits and climate change resilience

Here we find evidence supporting the potential for commercial exploitation to alter the diversity and distribution of metabolic phenotypes of fish populations, resulting in reduced magnitudes of aerobic scope and ability to maintain metabolic processes over a range of environmental temperatures. This reduced aerobic scope diversity and lack of high-performance aerobic scope phenotypes may have an impact on population level responses to future climate change events^19,20^. Our findings suggest that energy budget partitioning^41,42^ of exploited populations will have less capacity to adjust, especially when temperatures move towards the warmer limits of their thermal range. This has important ramifications for this species as marine heatwaves, which occur frequently throughout *C. laticeps*’ distribution^23^, are predicted to increase in frequency and severity globally as climate change progresses with time^2,24^.

The reduced aerobic scope and breakdown of SMR scaling with temperatures at the coldest experimental exposures are likely a result of individuals entering “cold-shock” following the stress of the acute decrease in temperature^43^. Despite the impact of extreme cold events on fish performance, such events have received relatively little attention in the climate change literature^44^. Our results highlight the potential negative impact and energetic constraints in response to abrupt cooling in both exploited and protected populations. The implications of cold shock may be particularly relevant for fish species in coastal intermittent upwelling zones, where intensification of upwelling favourable winds and associated cold temperature swings are predicted to continue increasing^45,46^. Yet the effects of climate change on coastal upwelling ecosystems remains relatively understudied^47^.

The reduced diversity of aerobic scope phenotypes is consistent with theory and research linking exploitation to a reduction in genetic diversity^48^ and functional trait diversity^49^. In an increasingly variable and uncertain climatic future, the diversity of physiological phenotypes is important to maintain the adaptive potential of populations^50^ and contribute to persistence across dynamic thermal contexts^14,20,51,52^.

### Mechanisms of selective exploitation

A key question is what mechanism is reducing the high-performance aerobic scope phenotypes in the exploited population. One possibility is a positive association between aerobic scope and behaviour that influences passive-gear fishing vulnerability. Indeed Redpath *et al.* (2010)^53^ showed that populations of largemouth bass, *Micropterus salmoides*, bred for high vulnerability to angling are associated with high aerobic scope phenotypes. While Hessenauer *et al*. (2015)^54^ showed that, in the wild, exploited populations of *M. salmoides* have reduced routine metabolic rates. Furthermore, aggression and boldness are behavioural types associated with a high vulnerability to capture in passive-gear fisheries^26,55,56^ and these behavioural types are often associated with high performance aerobic scope phenotypes^57–59^. Behavioural observations indicate high levels of intra-specific competition among *C. laticeps*^60^, which theoretically renders bold/dominant individuals more vulnerable to passive-gear fisheries. Our findings are thus in congruence with recent behaviour-based selection studies in fisheries that have shown that exploitation can reduce behavioural diversity of *M. salmoides*^61^, and can lead to fish populations with more timid individuals^62^.

While the most parsimonious explanation for our findings is the difference in fishing pressure between the two populations, other explanations are possible. For instance, different temperature signals or the condition of fish between the two sampling areas could lead to different metabolic profiles of the two populations. However, the temperature regime analysis indicated similar long-term trends and acute thermal changes between study areas and fish were evenly distributed among study area and temperature treatments in terms of mass and their condition. If factors such as food availability were markedly different between the two areas, we would expect a difference in the standard metabolic rate between the two populations – which we did not observe^63^. Furthermore, differences between populations were primarily due to maximum metabolic rates, which are less plastic and display little thermal compensation to acclimation temperatures^52^.

### Implications

Fishing – through the selective removal of specific phenotypic traits, can shape the evolutionary trajectory of exploited populations termed “fisheries induced evolution”^48,64^. Although previous research has primarily focused on how fisheries induced evolution can alter aspects of fish life history, such as growth rates^65^ or size at maturity and fecundity^66^, the theoretical groundwork for physiologically-based fisheries induced evolution in capture fisheries has recently been laid out^16^. For physiological-fisheries induced evolution to occur, physiological/metabolic phenotypes would need to be heritable, and certainly, growing evidence supports this contention^16,67^. For example, in populations of the Trinidadian guppy (*Poecilia reticulata*) metabolic rates have co-evolved with predation induced alterations to life history traits^68^. While this study has not specifically tested physiologically-based fisheries induced evolution it has shown how commercial exploitation in the wild can alter the metabolic trait distribution and diversity of fish populations. Given the heritability of metabolic traits our findings imply that humans do have the potential to alter the physiology of harvested fish populations – with implications on how they may respond to climate change.

Physiological traits shape the response of populations to the environment^14^, and thus research into fisheries selection pressures on physiological traits in the context of anthropogenic-induced climate change is emerging as an important area of conservation physiology research^16,37^. The potential for exploitation to change the distribution of physiological traits of fish populations further highlights the need for an evolutionary enlightened management approach in capture fisheries^48^ which can be achieved through spatial protection. At a population level, protection from fishing maintains larger populations that conserve age structures, life history parameters and maintain genetic diversity. Thus protection should enhance population stability by buffering the climate-recruitment relationship^6,69,70^ and maintain the raw material required for adaptation^71^.

This study extends the toolkit on how spatial protection can be implemented as a climate change management tool for fish populations, by harbouring populations with greater physiological phenotype diversity and high-performance aerobic scope phenotypes. Given the spill over effect of marine protected areas (MPA)^72,73^, well-designed networks of MPAs have the potential to buffer the selective removal of high-performance metabolic phenotypes in commercial fisheries, potentially leading to a greater level of physiological resilience of the population in a changing ocean.

## Methods

### Ethics

This research was conducted in accordance with the ethical regulations and approval from the Rhodes University Animal Ethics (DIFS152025) and the Animal Use and Care Committee of the South African National Parks (004/16).

### Study areas

Specimens from populations subjected to fisheries exploitation were obtained from the exposed side of the Cape Recife headland west of the Port Elizabeth (PE) metropolitan in South Africa (Fig. 1a,b). This area is heavily exploited by both the South African commercial linefishery (Fig. 1a) and a large recreational linefishery that launches from the nearby Noordhoek ski-boat club. Specimens from protected populations were obtained from within the Tsitsikamma National Park (TNP) Marine Protected Area (MPA) (Fig. 1a,b), which lies approximately 140 km west of PE. Tsitsikamma MPA was established as a no take MPA in 1964 and represents one of the  longest standing MPAs in the world where the status of fish stocks are considered pristine^74^.

### Thermal characteristics of study areas

The south coast of South Africa, experiences much greater variation in daily, monthly, and annual sea temperature compared to the west and east coasts (Figs 1d, 2)^23,75^. This is due to retention and cooling of Agulhas Current water on the Agulhas Bank, wind-driven upwelling and the effects of embayments and capes along this section of the coastline^23,76^. Extreme variability, driven by increases in marine heatwaves^23^ and cold spells, will most likely have the greatest biological impact along this coastline. Sea temperature-variability is also partly driven by the El Nino-Southern Oscillation (ENSO), where upwelling favourable easterly winds are strengthened during La Niña and weaken during El Niño^77^. Moreover, recent climate modelling consensus predicts an increase in the frequency and intensity of El Niño–Southern Oscillation (ENSO) events under the Paris Agreement’s global mean temperature increase targets^78–80^ and shifts in upwelling intensity, which will drive increases in associated sea temperature-variability along the South African coast^22^.

### Species profile

We selected the endemic seabream, *Chrysoblephus laticeps*, as a model species because it is heavily exploited by both the commercial and recreational ski-boat linefisheries in South Africa^81^, with catch estimates of between 10 000–100 000 kg recorded from locations (5 by 5 nautical mile grid cells) around PE (exploited area) between 1985–2017 and no reported catch from within the TNP MPA (protected area)^82^ (Fig. 1a,c). The commercial and recreational ski-boat linefisheries are multispecies fisheries that use passive hook and line as the capture method, with around 455 commercial boats operating in South Africa up until 2013^83^. *Chrysoblephus laticeps* is also extremely resident, with the probability of being recaptured within the marine reserve estimated at 0.94^40^. Using acoustic telemetry data its home range size was estimated to be small; between 1000–3000 m^2 ^^60^. Furthermore *C. laticeps* is not genetically structured into multiple evolutionary distinct populations that may have evolved different strategies^84^. We expect our findings to be generalizable because the life history and behavioural characteristics of C. *laticeps* mirror other demersal reef species that form part of the multispecies linefishery in South Africa.

### Collection and husbandry

Live *C. laticeps* specimens were caught using hook and line off ski-boats in water no deeper than 25 m from exploited (n = 25) and protected (n = 25) areas. Predetermined fishing marks were selected in each area where reef patches are known to occur. After arriving at a mark, line fishing began, while the ski-boat drifted over the reef patch. If the ski-boat drifted off the reef-patch we travelled to a new mark and continued fishing. Marks were randomly chosen throughout suitable areas at each site and fishing effort was standardised in terms of fishing gear. Once landed, fish were vented using a hypodermic needle and placed into a 1000 L tank filled with fresh seawater, transported back to the shore and transferred to a large, circular holding tank, which was continuously supplied with fresh seawater using a submersible pump. Live specimens were subsequently transported to a laboratory where they were acclimated for 6 weeks at 16 °C in a recirculating holding facility. Photoperiod was set to 9.5 h of light and 14.5 h dark and the water parameters; oxygen, salinity, pH and ammonia checked daily ensuring they never fell outside natural ranges. Fish were fed a diet consisting of frozen sardine (*Sardinops sagax*) and squid (*Loligo reynaudii*) every other day, ensuring every fish ingested food, and no individuals lost weight.

### Experimental procedure

Fish were starved for 36 h prior to experimentation and placed into a custom-built 30.8 L intermittent flow respirometer at 16 °C. Fish were allowed to acclimate to the respirometer for 12 h, after which one of the temperature-variability simulations was started. In all simulations, the temperature was modified at a rate of ~1 °C per h until an endpoint of 8, 12, 16 (no modification), 20 or 24 °C was reached. The endpoint temperature was then maintained for ~22 h to determine standard metabolic rate using intermittent flow respirometry (15/17 min flush – 5/3 min measure). To elicit maximum metabolic rate, individuals were transferred to an 800 l circular tank, chased to exhaustion for 10 min and exposed to air for 30 s before they were returned to the respirometer. Intermittent flow respirometry was then resumed immediately for ~4 h until metabolic rates reduced towards levels close to SMR. Background respiration rates were calculated after each trial in an empty respirometer for ~3 h. Each fish was included in only one experiment, and test temperatures and respirometers were randomized throughout the experiment.

### Metabolic rate measurements

A quality threshold R^2^ > 0.9 was implemented to filter the linear decline in oxygen during measurement periods except for the 8 °C test temperature, where a threshold of 0.8 was used to maintain sample sizes as rates of oxygen decline were low. Whole-organism metabolic rate was calculated using the equation developed by^85^ for each measurement period (Eq. 1).

1 $$R{{\rm{O}}}_{2}=((\frac{{{\rm{V}}}_{{\rm{re}}}-{\rm{M}}}{{\rm{W}}})(\frac{{\rm{\Delta }}[{{\rm{O}}}_{2{\rm{a}}}]}{{\rm{\Delta }}t}\times {\rm{60}}))-((\frac{{{\rm{V}}}_{{\rm{re}}}-{\rm{M}}}{{\rm{W}}})(\frac{{\rm{\Delta }}[{{\rm{O}}}_{{\rm{2b}}}]}{{\rm{\Delta }}t}\times {\rm{60}})(\frac{{{\rm{V}}}_{{\rm{re}}}}{({{\rm{V}}}_{{\rm{re}}}-{\rm{M}})}))$$

where *V*_*re*_ is the total volume of the respirometer in litres; *M* is the mass of the specimen in kg expressed  in l; W is mass of the specimen in kg, $\frac{{\rm{\Delta }}[{{\rm{O}}}_{2{\rm{a}}}]}{{\rm{\Delta }}t}$ is the slope of the linear decrease in oxygen concentration during the measurement period and $\frac{{\rm{\Delta }}[{{\rm{O}}}_{2{\rm{b}}}]}{{\rm{\Delta }}t}$ is the slope of the linear decrease in oxygen concentration when no specimen was in the chamber (background respiration).

Standard metabolic rate was calculated as the 0.2 quantile of all metabolic rate measurements at test temperatures prior to the elicitation of MMR measurements^32^. Maximum metabolic rate was determined during excess post-exercise oxygen consumption as the single biggest metabolic rate measurement. To mass standardise, metabolic rate data were multiplied by the Boltzmann factor to correct for temperature effects with the activation energy taken as the average activation energy of ectotherms^86^ (Eq. 2).

2 $$R{{\rm{O}}}_{2(\mathrm{temp}\mathrm{corrected})}=R{{\rm{O}}}_{2}\times {{e}}^{\frac{-{E}}{{kT}}}$$

where *E* is the average activation energy of ectotherms ~0.63 eV^86^, *k* is the Boltzmann constant 8.617 333 × 10^−5^ eV.K^−1^ and *T* is the absolute temperature in kelvin.

Metabolic rate data was mass corrected by dividing by the allometric mass-scaling relationship (Eq. 3), derived from the regression of the natural logarithms of temperature corrected metabolic rates against mass (see Supplementary Material Figs SM4a–c and SM5a–c).

3 $$M{{\rm{O}}}_{2}=\frac{R{{\rm{O}}}_{2}}{{M}^{\alpha }}$$

where *M*O_2_ is mass normalised SMR or MMR, *R*O_2_ is standard or maximum oxygen consumption rate, *M* is the mass of the organism and α is the allometric mass scaling exponent.

Individual absolute aerobic scope was calculated as the difference between mass-corrected MMR and SMR.

### Statistical analysis

A generalised least squares (GLS) modelling approach was implemented using the nlme package^87^ to account for data heteroscedasticity. Differences in mass-corrected metabolic data between exploited and protected areas were tested by modelling a second order polynomial relationship between metabolic data and temperature (adjusted so coldest test temperature (8 °C) corresponded to model intercept) including site as an interaction term and a variance structure weighted by temperature and site. Orthogonal polynomials were used for statistical inference to reduce the effect of collinearity among explanatory polynomial terms^88^. Metabolic phenotype variability was compared using a paired (by test temperature) one-way student’s t-test on the variance structure of the aerobic scope GLS model. To contextualise the physiological performance limit in response to future extreme upwelling events we plotted the relationship between the natural logarithm of standard metabolic rate against the inverse product of temperature and the Boltzmann constant and fit a piece-wise linear breakpoint relationship using the segmented package^89,90^.

### Study area and test specimen comparison

Because thermal history can shape metabolic profiles^91,92^ we also tested if the two study areas (protected and exploited) had similar long-term trends and short-term variability patterns in sea temperature. Furthermore, metabolic rates are influenced by characteristics such as mass and condition. We thus ensured that test specimens were evenly distributed across treatments and test temperatures and that mass effects were adequately accounted for.

### Sea temperature data

Long-term (1981–2015) REYNOLDS AVHRR V2 high precision OI daily sea surface temperature (SST) data for protected and exploited areas were obtained from the National Oceanic and Atmospheric Administration’s National Climate Data Centre website: http://iridl.ldeo.columbia.edu/SOURCES/.NOAA/.NCDC/.OISST/.version2/.AVHRR^93^. Level-2 SST data captured by the Moderate Resolution Imaging Spectroradiometer (MODIS) aboard the Terra satellite for the South African Coast on 04-03-2010 were obtained from the Ocean Colour website: http://oceancolor.gsfc.nasa.gov (NASA Goddard Space Flight Center). MODIS-Terra data was reprocessed at a 4 km resolution and masked with the CLDICE flag using the Data Analysis System (SeaDAS)^94^. Underwater Temperature Recorder (UTR) daily sea temperature data were obtained from the Southern African Data Centre for Oceanography (SADCO) for the Tsitsikamma Marine Protected Area (10 m depth) and Mangolds Pool near Port Elizabeth (5 m depth) deployments. Only concurrent data from both UTRs were included in the analysis. Hourly thermistor string data from within the Tsitsikamma National Park at depths of 12, 19, 27 and 35 m were obtained from SADCO.

### Thermal regime analysis

To compare long-term sea temperature trends between exploited and protected areas the daily AVHRR SST data was modelled using a generalized additive mixed effects model (GAMM) including a seasonal cyclical smoothing spline and a trend smoothing spline for each area with an autoregressive moving average (1,0,0) correlation structure to remove autocorrelation, in the mgcv package^95^. The difference between the exploited and protected trend smoothing splines was tested for significance from zero using ordered factors within the GAMM. While remotely sensed SST data can be useful when exploring large-scale, long-term, temporal patterns it can sometimes fail to capture localised SST variability on shorter time scales (e.g. upwellings) when data points are located close to shore^75^. Because the South African south coast is characterised by localised variability in sea temperature it was important to investigate the similarity of this variability between areas, which was done using higher resolution UTR time series data. Each time series was decomposed into its trend, seasonal and random components using the tseries package^96^. The random component of each UTR time series is irregular variance that can represent intermittent sea temperature-variability such as upwelling/downwelling events^97^. The similarity in direction and magnitude of irregular sea temperature-variability between exploited and protected areas was tested for significant synchrony using the meancorr function in the synchrony package^98^ where spatial and temporal correlation were removed by a naïve randomization procedure and tests for significant correlations were performed via 999 Monte Carlo randomizations. To illustrate the ecological applicability of acute thermal changes, hourly thermistor string data (19 m depth) were plotted as a time series and rates of increase and decrease in temperature-variability were explored.

We tested for differences in mass and condition factor ((mass × 10^5^)/(FL^3^), where mass is in grams and FL is fork length in mm) between protected and exploited specimens using one tailed T tests. We visually inspected the distribution of mass standardised aerobic scope phenotypes against mass and mass distributions of specimens against test temperatures per exploited and protected areas to ensure evenness.

## Supplementary information

Supplementary results

## Data Availability

Data supporting the main findings, figures and analysis of this study are available from the corresponding author upon request.
