# Supplementary material for: Exploitation may influence the climate resilience of fish populations through removing high performance metabolic phenotypes
Source: Sci Rep. 2019 Aug 7;9:11437. doi: 10.1038/s41598-019-47395-y (PMC6685998; doi:10.1038/s41598-019-47395-y)
Supplement: Supplementary file 1 — Supplementary results [file 41598_2019_47395_MOESM1_ESM.docx]

**Supplementary Information**

**Exploitation may influence the climate resilience of fish populations through removing high performance metabolic phenotypes**

Murray I. Duncan*^1,2^, Amanda E. Bates^3^, Nicola C. James^2^, Warren M. Potts^1^

1: Department of Ichthyology and Fisheries Science, Rhodes University, Grahamstown, 6140, South Africa

2: South African Institute for Aquatic Biodiversity, Grahamstown, 6139, South Africa

3: Department of Ocean Sciences, Memorial University of Newfoundland, St. John’s A1C 5S7, Canada

*: corresponding author, email: [muzz.duncan@gmail.com](mailto:muzz.duncan@gmail.com)

**Supplementary Data tables and figures**

**
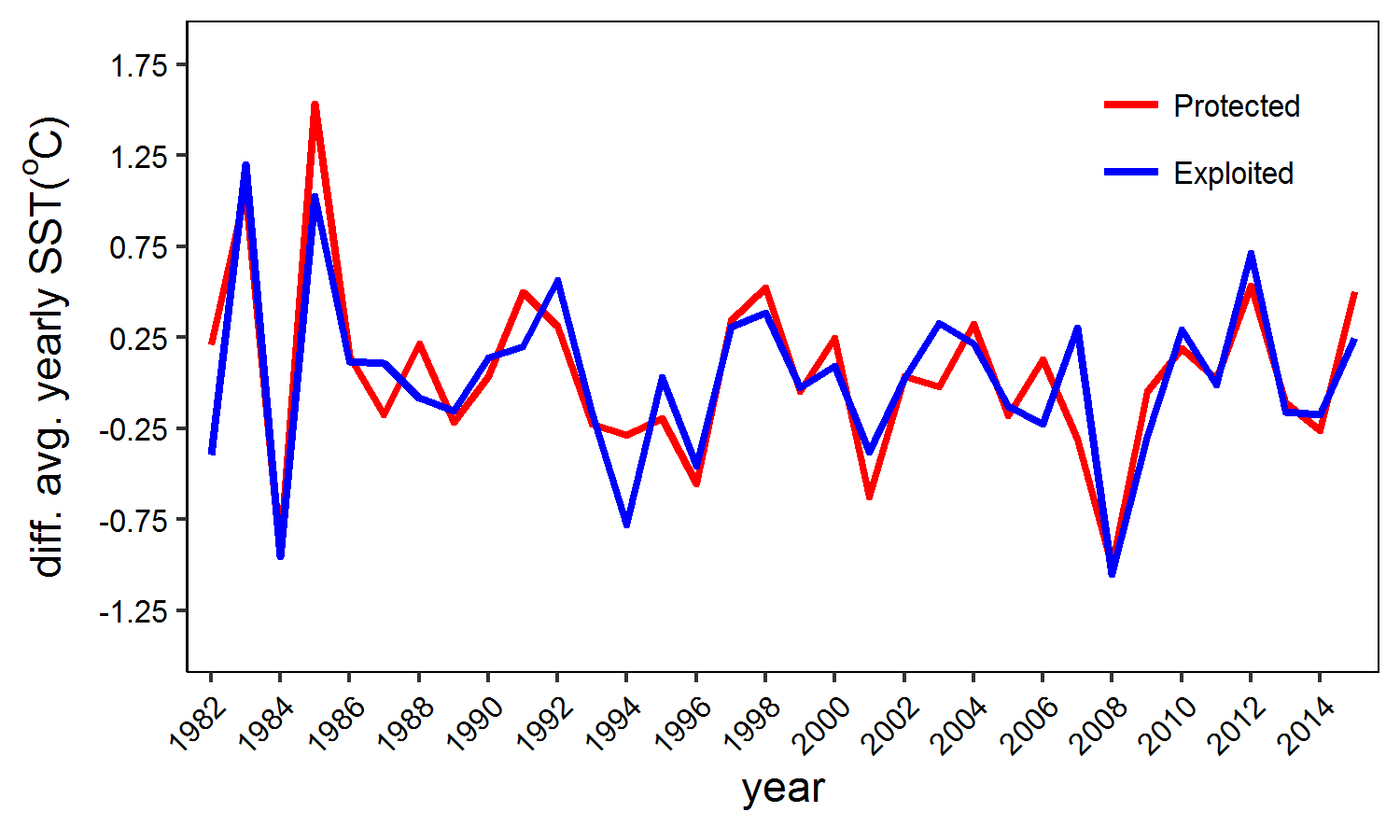
**

(b)

(a)

**
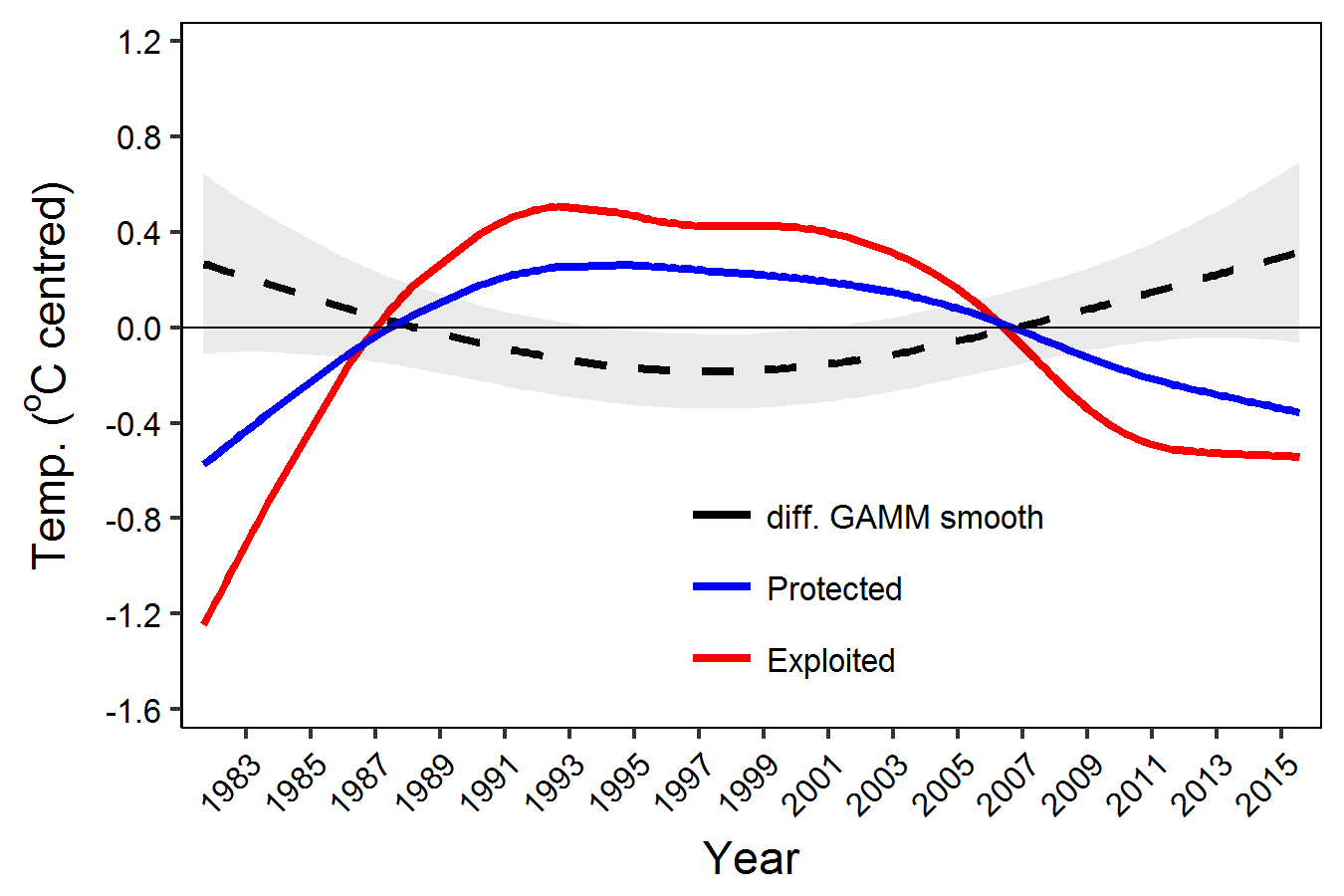
**

**Supplementary Figure SM1. Long term SST trend between areas. a.** Year to year differences in mean annual SST derived from AVRR satellite data, **b.** GAMM modelled SST trend splines including the difference in SST trend spline (dashed black line with 95% confidence intervals shaded in grey)

**
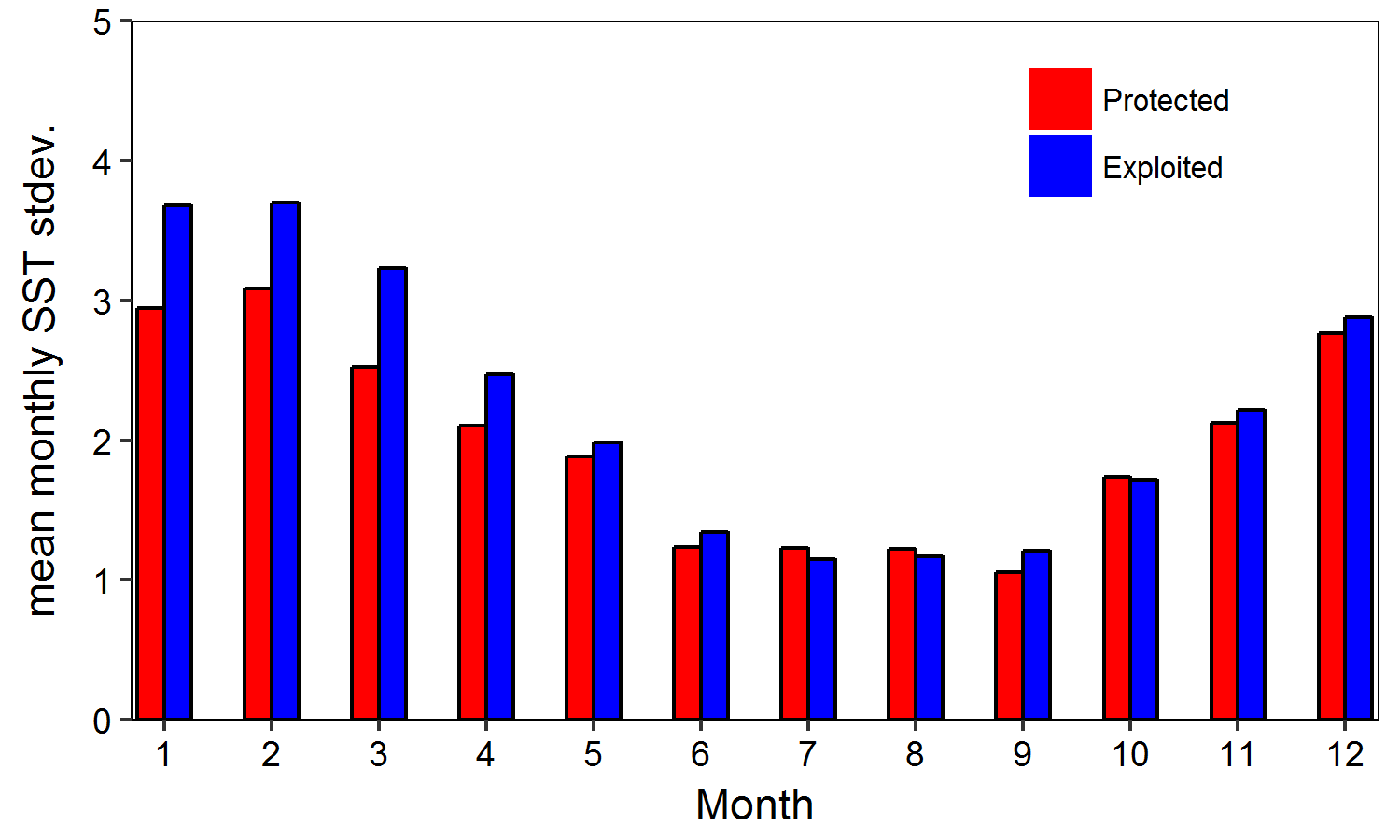
**

(b)

(a)

**
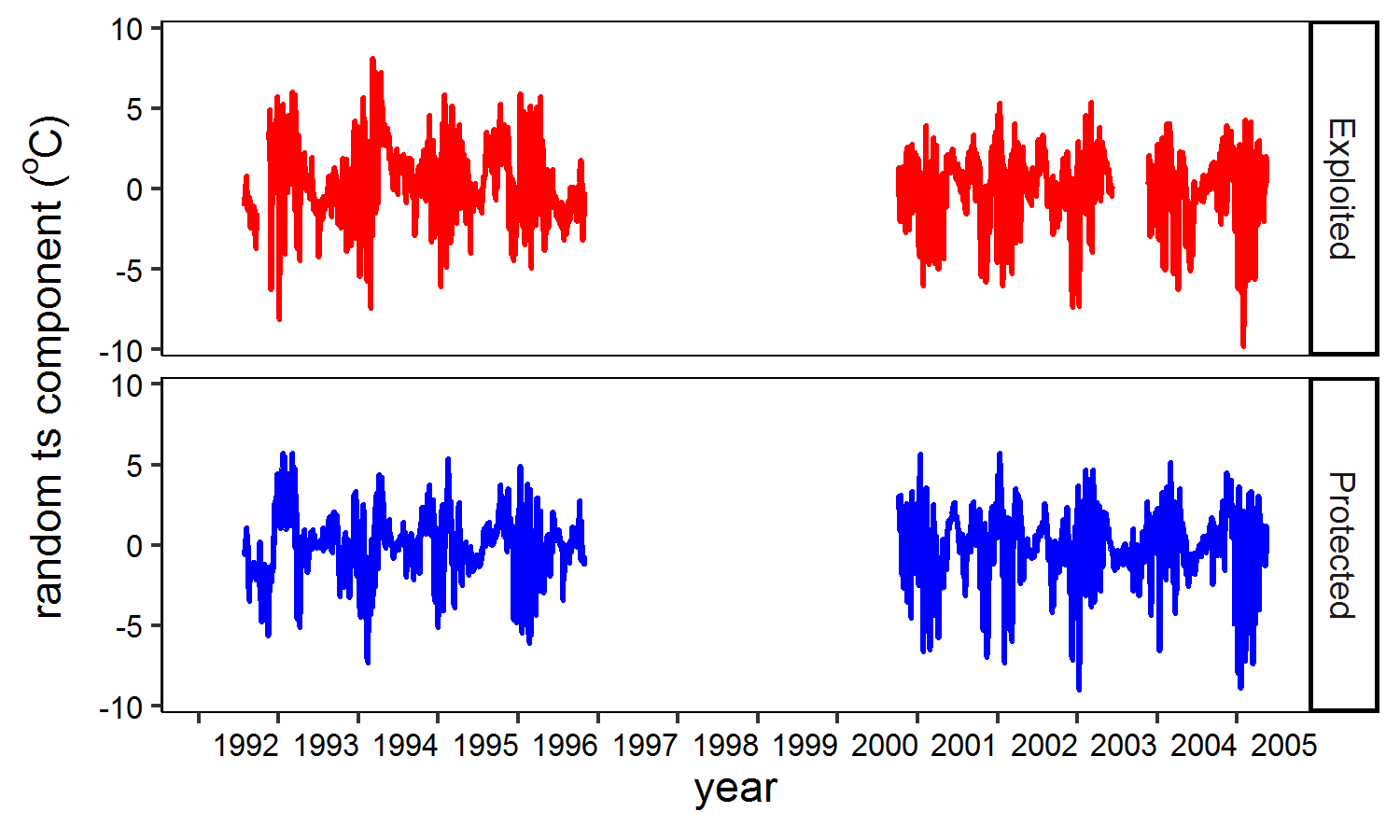
**

(c)

**
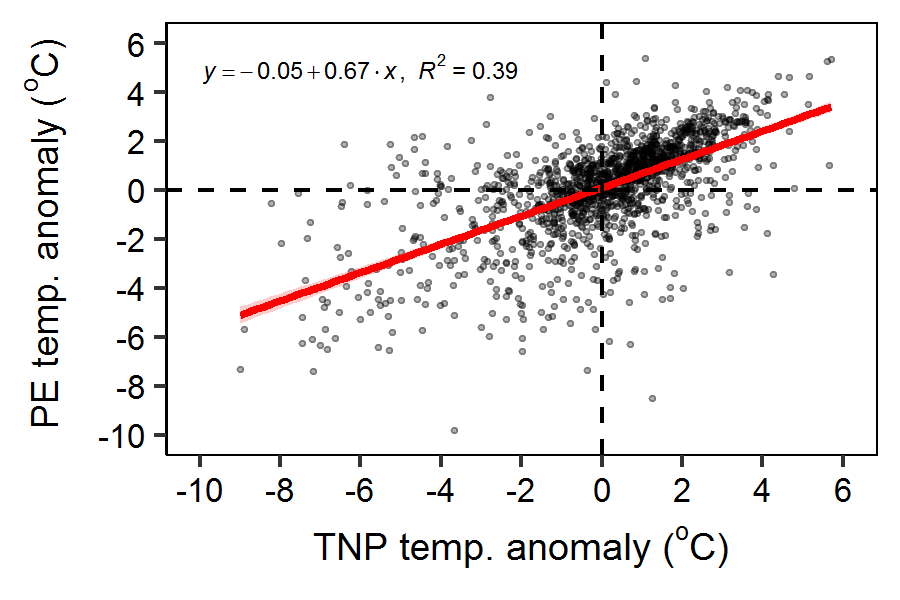
**

**Supplementary Figure SM2. Short term sea temperature variability regimes. a.** Standard deviation of monthly mean UTR sea temperature data for exploited (red- 5m) and protected (blue – 10m) sampling areas. **b.** Time series of the random component of UTR data (random time series component) exploited (red – 5m) and protected (blue – 10m) sampling areas. **c.** Linear correlation between temperature anomalies (random component of UTR data) for sampling areas.


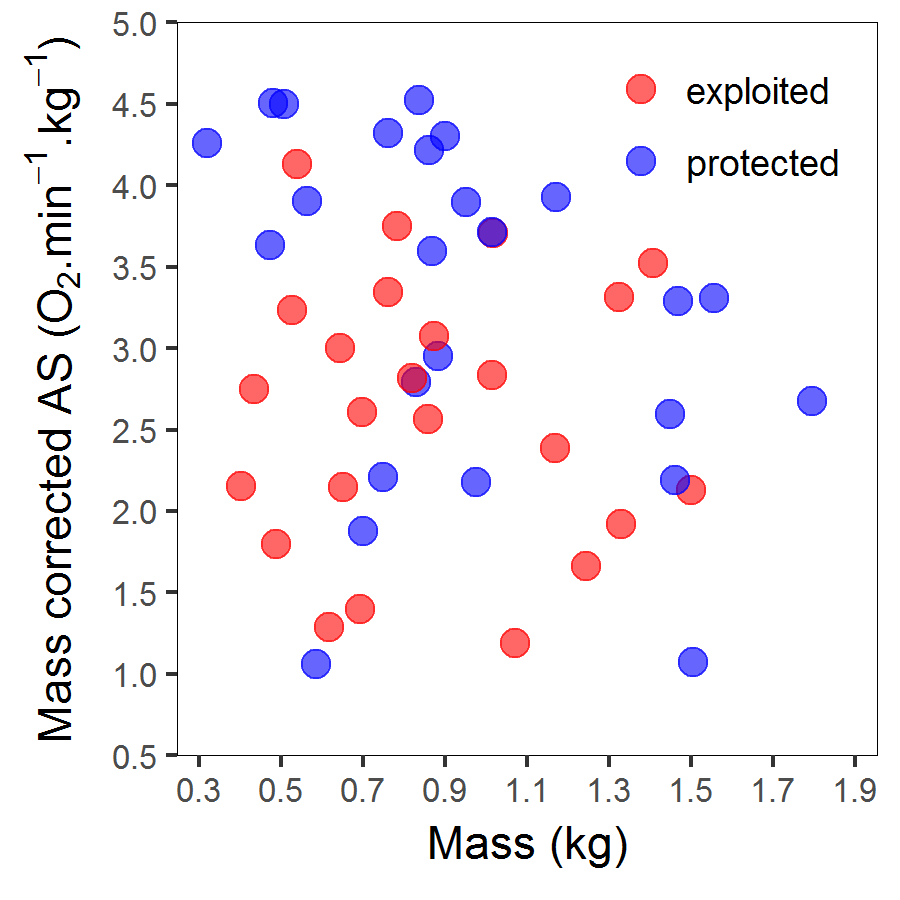

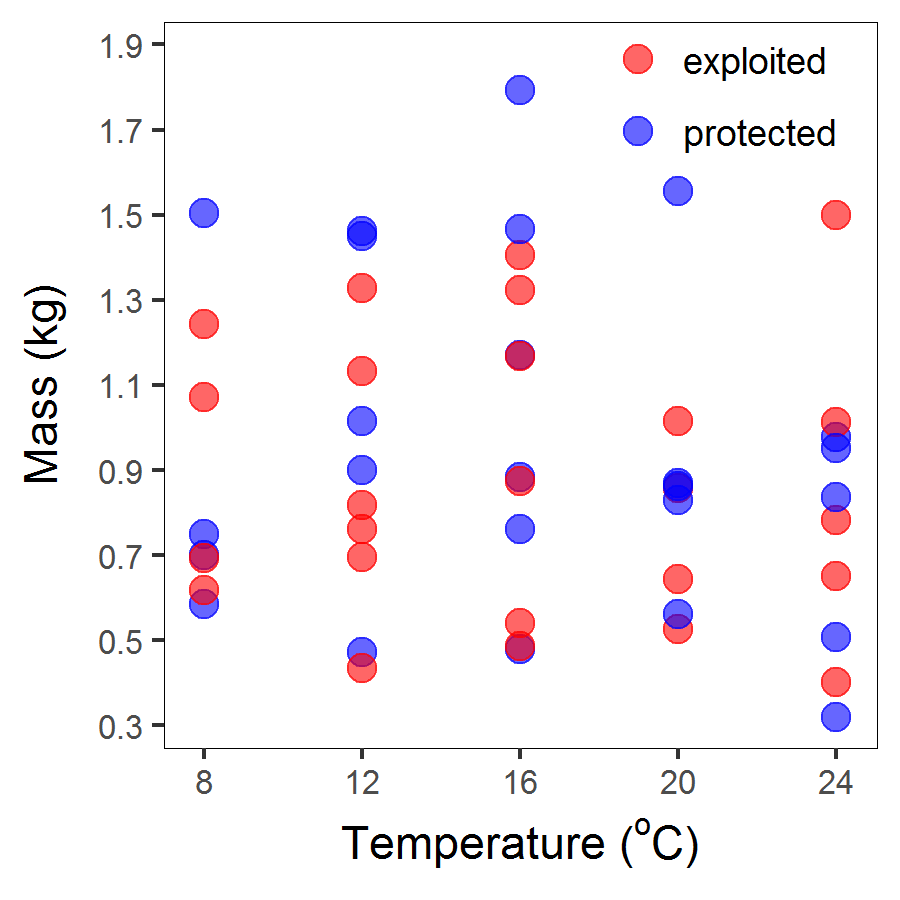


(b)

(a)

**Supplementary Figure SM3. Distribution of specimen masses for study. a.** Mass corrected aerobic scope (AS) vs original mass (points) for exploited (red) and Protected (blue) specimens. **b.** Mass of specimen’s vs test temperature (points) for exploited (red) and Protected (blue)

| 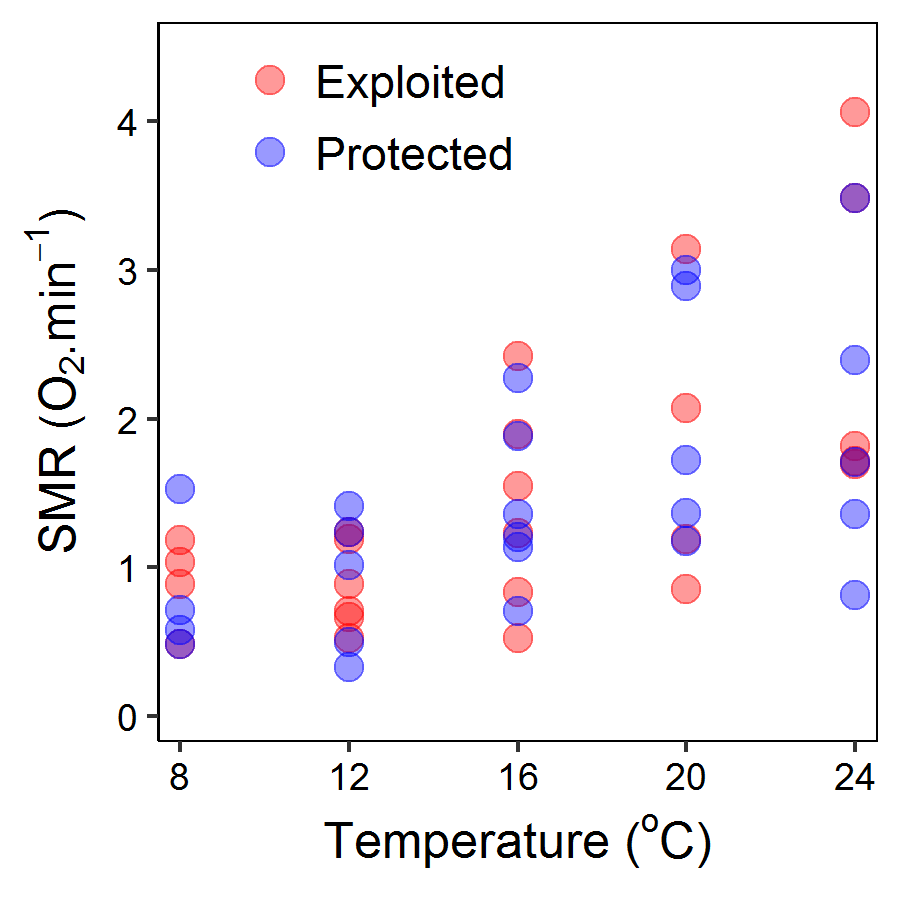  a) | 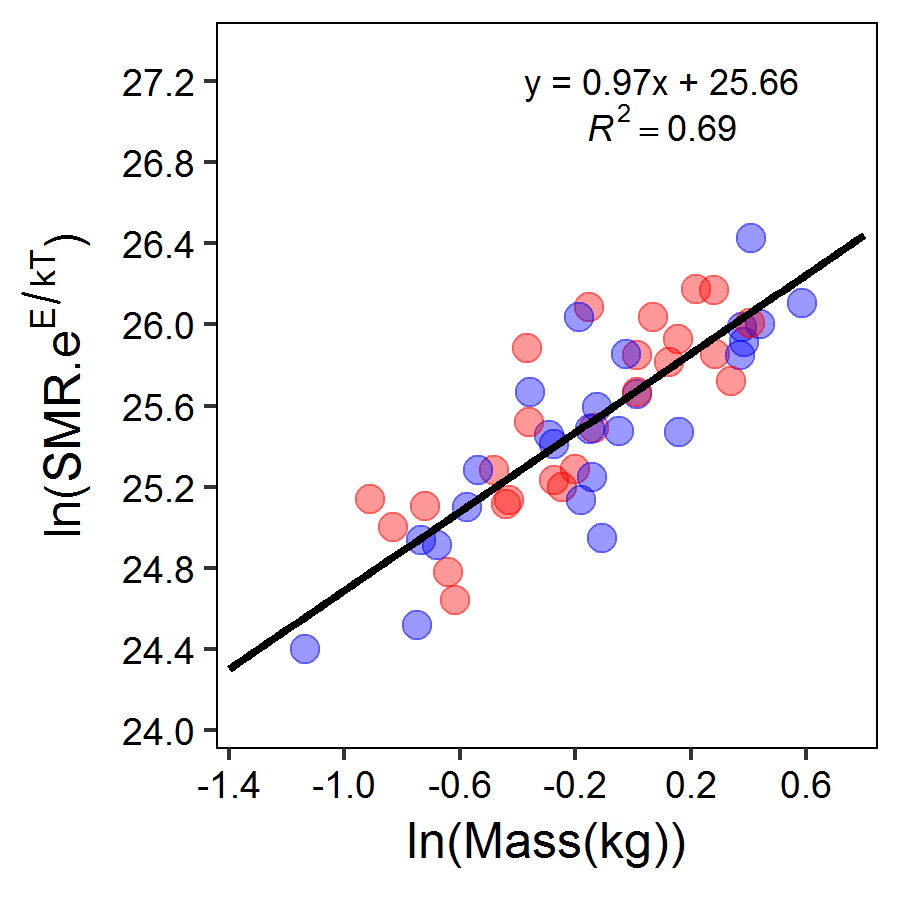  b) |
| --- | --- |
| 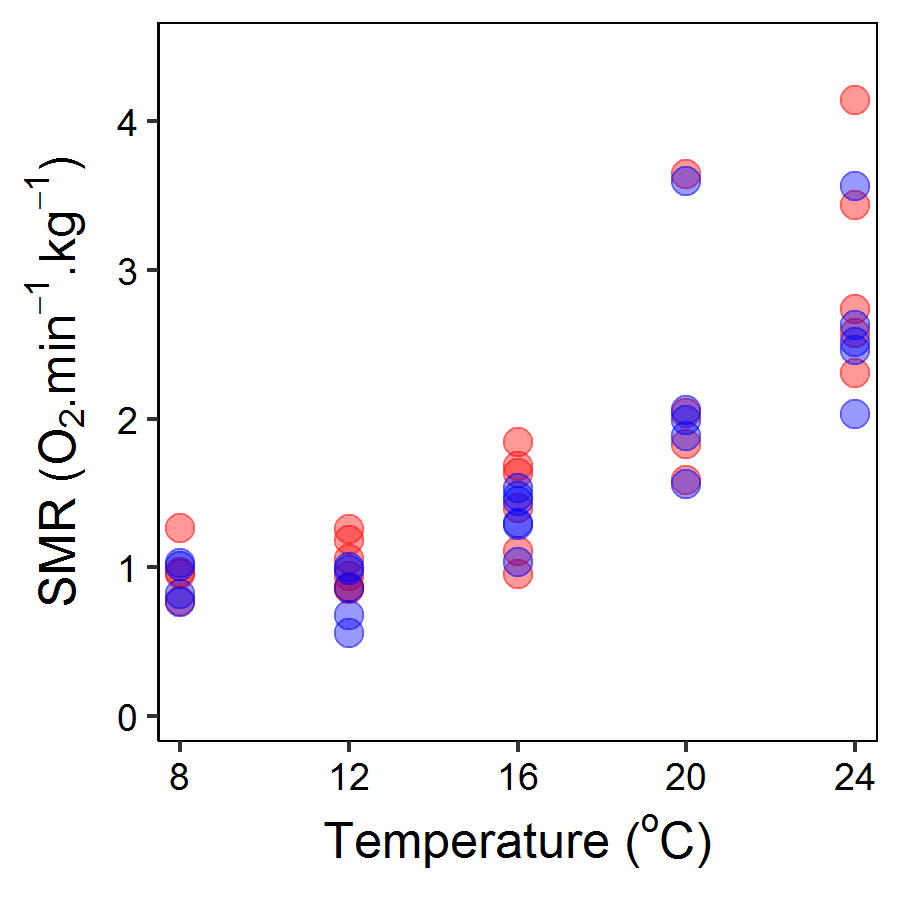  c) |  |

**Supplementary Figure SM4. Mass correcting SMR. a.** Raw SMR data (SMR (O_2_.min^-1^)) per temperature, **b.** regression of the natural logarithm of temperature-corrected SMR (*ln*(SMR.e^E/kT^)) against the natural logarithm of mass (ln(Mass(kg)), **c.** and mass-corrected SMR data (SMR (O_2_.min^-1^)) per temperature used for the analysis.

| 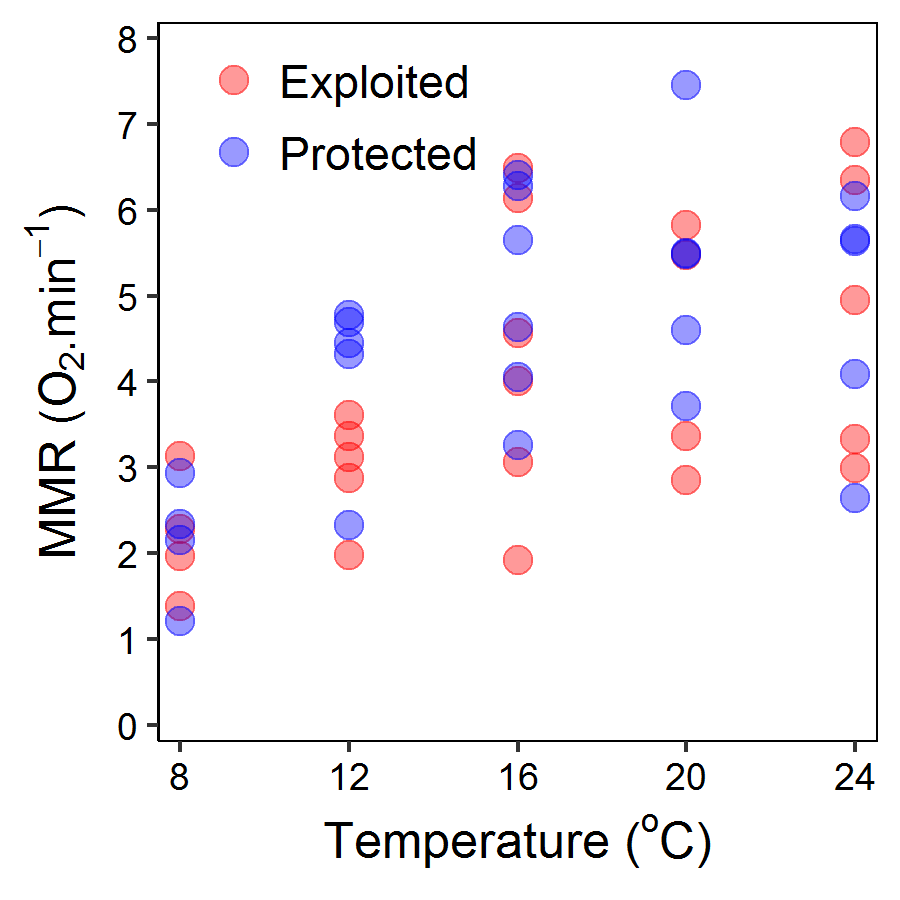  a) | 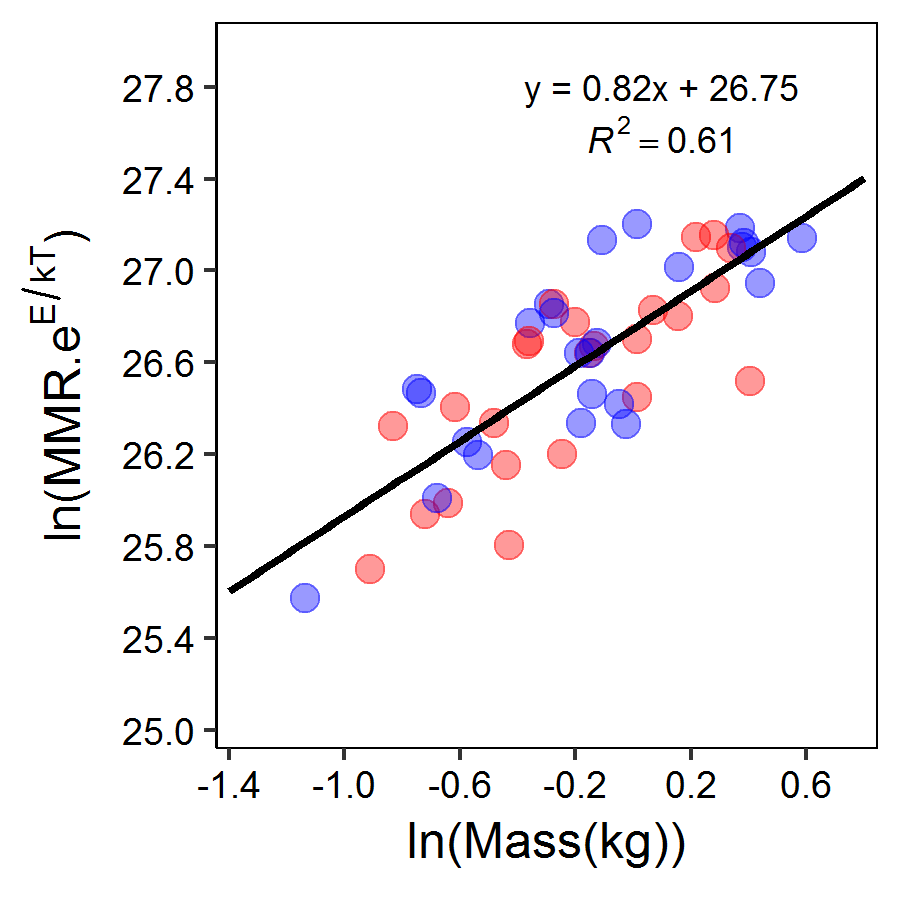  b) |
| --- | --- |
| 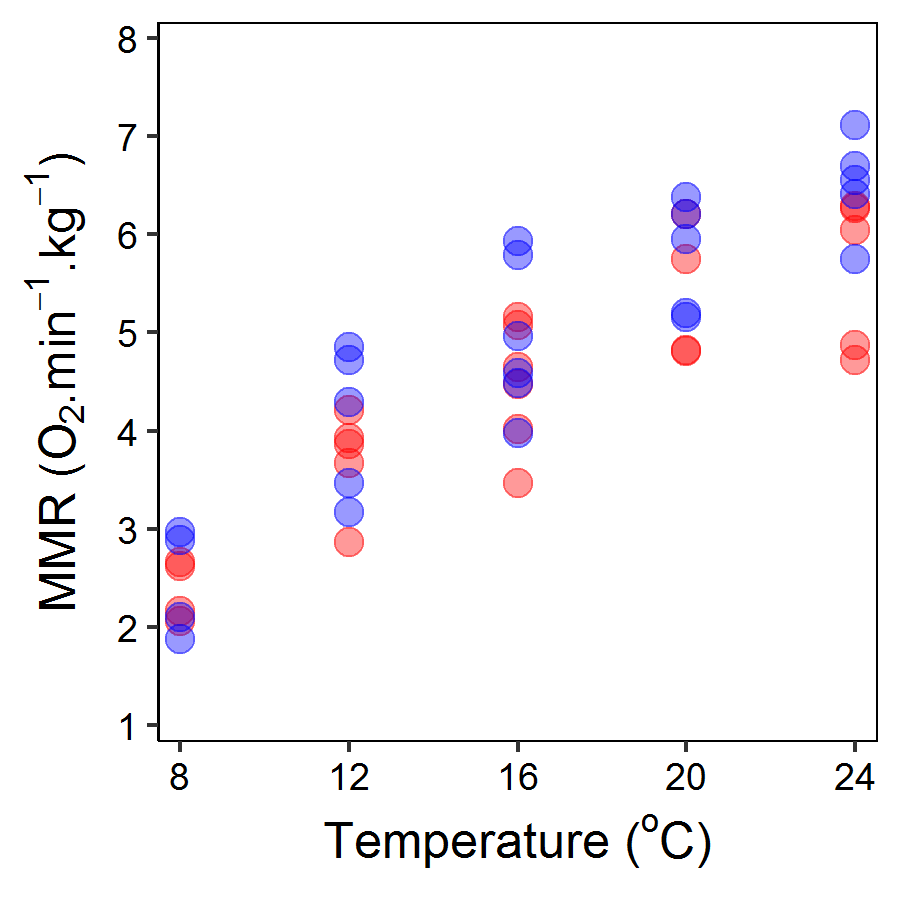  c) |  |

**Supplementary Figure SM5. Mass correcting MMR data. a.** Raw MMR data (MMR (O_2_.min^-1^)) per temperature, **b.** regression of the natural logarithm of temperature-corrected MMR (*ln*(MMR.e^E/kT^)) against the natural logarithm of mass (ln(Mass(kg)) **c.** and mass-corrected MMR data (MMR (O_2_.min^-1^)) per temperature used for the analysis.

**Supplementary Table SM1.** Generalised least squares modelling results of metabolic rate as a second order polynomial function of temperature with site as an interaction term. SE is standard error, AIC is Akaike information criterion, DF is total degrees of freedom and significant p-values are highlighted in bold. **a.** Standard metabolic rate. **b.** Maximum metabolic rate. **c.** Absolute aerobic scope

**(a)**

| Effect | Estimate | SE | t-value | p-value |
| --- | --- | --- | --- | --- |
| Intercept | 1.727 | 0.098 | 17.674 | **0.000** |
| Site | -0.154 | 0.122 | -1.265 | 0.212 |
| Temp | 5.286 | 0.757 | 6.978 | **0.000** |
| Temp^2^ | 1.856 | 0.508 | 3.654 | **0.001** |
| Site:temp | -0.495 | 0.982 | -0.504 | 0.617 |
| Site:temp^2^ | -0.246 | 0.654 | -0.376 | 0.709 |
|  |  |  |  |  |
| AIC | 65.464 |  |  |  |
| Residual SE | 0.201 |  |  |  |
| DF | 49 |  |  |  |

**(b)**

| Effect | Estimate | SE | t-value | p-value |
| --- | --- | --- | --- | --- |
| Intercept | 4.370 | 0.121 | 36.222 | **0.000** |
| Site | 0.437 | 0.173 | 2.528 | **0.015** |
| Temp | 7.715 | 0.839 | 9.193 | **0.000** |
| Temp^2^ | -1.689 | 0.771 | -2.191 | **0.034** |
| Site:temp | 1.589 | 1.140 | 1.394 | 0.170 |
| Site:temp^2^ | 0.216 | 1.133 | 0.191 | 0.849 |
|  |  |  |  |  |
| AIC | 111.392 |  |  |  |
| Residual SE | 0.307 |  |  |  |
| DF | 49 |  |  |  |

**(c)**

| Effect | Estimate | SE | t-value | p-value |
| --- | --- | --- | --- | --- |
| Intercept | 2.626 | 0.108 | 24.327 | **0.000** |
| Site | 0.515 | 0.179 | 2.852 | **0.007** |
| Temp | 2.451 | 0.728 | 3.365 | **0.002** |
| Temp^2^ | -3.491 | 0.745 | -4.683 | **0.000** |
| Site:temp | 1.737 | 1.349 | 1.288 | 0.205 |
| Site:temp^2^ | 0.694 | 1.321 | 0.525 | 0.602 |
|  |  |  |  |  |
| AIC | 116.801 |  |  |  |
| Residual SE | 0.204 |  |  |  |
| DF | 49 |  |  |  |

**Supplementary Table SM2.** Approximate significance of smooth terms from the GAMM model to compare SST trend splines between sampling areas. Significant p-values are highlighted in bold.

| Smooth term | edf | F-value | p-value |
| --- | --- | --- | --- |
| Seasonal spline exploited | 7.792 | 61.869 | **<0.001** |
| Seasonal spline protected | 8.724 | 86.036 | **<0.001** |
| Reference trend spline | 12.099 | 11.243 | **<0.001** |
| Difference trend spline | 2.082 | 3.077 | 0.052 |

**Supplementary Table SM3.** Weights and condition factor (CF) per sampling area of specimens used in this study. There was no significant difference in condition factor or mass of specimens between sampling areas (one tailed T test, *p* value > 0.05).

| area | n | CF mean | mass mean (g) | mass range (g) |
| --- | --- | --- | --- | --- |
| exploited | 25 | 2.86 | 879 | 402 - 1500 |
| protected | 25 | 2.84 | 946 | 320.9 - 1794 |
